# Impact of insurance status on ability to return for outpatient management of pediatric supracondylar humerus fractures

**DOI:** 10.1007/s11832-016-0769-x

**Published:** 2016-08-25

**Authors:** Nicholas D. Fletcher, Bryan J. Sirmon, Ashton S. Mansour, William E. Carpenter, Laura A. Ward

**Affiliations:** 1 Department of Orthopaedics, Emory University, 59 Executive Park South NE, Atlanta, GA 30329 USA; 2 Department of Biostatistics, Rollins School of Public Health, Emory University, 1518 Clifton Rd, Atlanta, GA 30322 USA

**Keywords:** Supracondylar humerus fracture, Insurance, Medicaid, Access to care

## Abstract

**Purpose:**

Outcomes are excellent following surgical management of displaced supracondylar humerus fractures. Short delays until surgical fixation have been shown to be equivalent to immediate fixation with regards to complications. We hypothesized that insurance coverage may impact access to care and the patient’s ability to return to the operating room for outpatient surgery.

**Methods:**

A retrospective review of supracondylar humerus fractures treated at a large urban pediatric hospital from 2008 to 2012 was performed. Fractures were classified by the modified Gartland classification and baseline demographics were collected. Time from discharge to office visits and subsequent surgical fixation was calculated for all type II fractures discharged from the emergency department. Insurance status and primary carrier were collected for all patients.

**Results:**

2584 supracondylar humerus fractures were reviewed, of which 584 were type II fractures. Of the 577 type II fractures with complete records, 383 patients (61 %) were admitted for surgery and the remaining 194 were discharged with plans for outpatient follow-up. There was no difference in insurance status between patients admitted for immediate surgery. Of the 194 patients who were discharged with type 2 fractures after gentle reduction, 59 patients (30.4 %) ultimately underwent surgical fixation. Of these, 42 patients were privately insured (58.3 % of patients with private insurance), 16 had governmental insurance (15.1 %), and 1 was uninsured (6.3 %). Patients with private insurance were 2.46 times more likely to have surgery than patients with public or no insurance (*p* = 0.005). Of the 135 patients who did not eventually have surgery, 92 (68.1 %) were seen in the clinic. Patients with private insurance were 2.78 times more likely to be seen back in the clinic when compared to publicly insured or uninsured patients (*p* = 0.0152).

**Conclusions:**

Despite an equivalent number of privately insured and publicly insured patients undergoing immediate surgery for type II fractures, those with public or no insurance who were discharged were 2.46 times less likely to obtain outpatient surgery when compared to privately insured patients. Patient insurance status and the ability to follow up in a timely manner should be assessed at the time of initial evaluation in the emergency department.

*Level of evidence* Level 3

## Introduction

Pediatric supracondylar humerus fractures are the most common pediatric fracture of the elbow. While operative management remains the standard of care for Gartland type III fractures, controversy persists regarding the optimum treatment for Gartland type II fractures [1]. Treatment options include closed reduction with either casting or percutaneous pinning [2]. The American Academy of Orthopaedic Surgeons (AAOS) has published in its clinical practice guidelines for the treatment of pediatric supracondylar humerus fractures that “closed reduction with pin fixation [is suggested] for patients with displaced (Gartland type II and III, and displaced flexion) pediatric supracondylar fractures of the humerus” [3]. Multiple studies have found no difference in complication rates between early versus delayed treatment of a type 2 fracture pattern, allowing outpatient surgery and potentially lower costs to the healthcare system [4–6]. Closed treatment with reduction and casting may also result in excellent radiographic and clinical outcomes assuming early follow-up and no loss of reduction [7–9]. Regardless of the definitive treatment, limitations in access to care can jeopardize the feasibility of a delayed surgery, as patients requiring close follow-up may not be able to obtain access to a provider in time to have surgery. This study aims to evaluate the subset of patients with type II supracondylar humerus fractures with either no insurance or government/public insurance, and whether they suffered a lapse or loss in care due to their insurance status versus patients with private insurance.

## Methods

After approval by the institutional review board, a retrospective review was performed of patients with isolated, unilateral Gartland type II supracondylar humerus fractures who were treated within the emergency departments at two metropolitan children’s hospitals between 2008 and 2012 by surgeons in four pediatric orthopedic practices. A total of 2619 patients were identified during this time period with the ICD-9 code 812.41, correlating to a fracture of the supracondylar region of the humerus. Patients with ipsilateral forearm or wrist injuries were excluded, leaving a total of 2583 patients with isolated injuries to the supracondylar humerus. These fracture types were identified and classified according to the modified Gartland classification [10, 11]. The dictated operative notes from the attending staff were used as the definitive classification. As this study focused on fractures treated in a delayed fashion, a formal radiographic review of patients admitted from the ED for operative fixation was not performed, based on the assumption that surgery would not be performed on nondisplaced (type 1) fractures. All patients with clinically classified type 2 fractures who were discharged from the ED underwent radiographic review to confirm the classification seen in the chart. Insurance status at the time of initial presentation was determined from the electronic medical record. Time to surgery was determined as the period from the date of the initial evaluation of the injury in the emergency department to the date of any surgical intervention for the initial injury.

## Statistical analysis

Counts and frequencies were tabulated for insurance type and by clinic status. A chi-square test was used to check the statistical relationship between insurance and status. A generalized estimating equation analysis was completed to examine the statistical significance of the differences in overall surgery and immediate surgery between insurance groups while controlling for any correlation that may exist between patients who suffered a second ipsilateral or contralateral fracture at a remote timepoint within the inclusion period. A mixed means model was used to calculate the adjusted mean time to surgery and compare it between private and non-private insurance groups, considering all patients or only those who had a delayed surgery.

## Results

2583 supracondylar humerus fractures were reviewed. There were 1134 type 1 (43.8 %), 583 type 2 (22.6 %), and 866 type 3 (34 %) fractures (Table 2). 1508 patients (58 %) were identified as having private payor insurance. 919 patients (36 %) had public insurance and 156 (6 %) patients were uninsured (Table 1).

**Table 1:** Patient demographics for all patients with supracondylar humerus fractures

Age (years) (*n* = 2583)	
0–4	1088 (42 %)
4–8	1199 (46 %)
8–12	250 (10 %)
>12	46 (2 %)
Sex (*n* = 2853)	
Female	1203 (47 %)
Male	1380 (53 %)
Insurance status (*n* = 2583)	
Private	1508 (58 %)
Public	919 (36 %)
Uninsured	156 (6 %)
Insurance status of type 2 patients (*n* = 583)	
Private	313 (54 %)
Public	247 (42 %)
Uninsured	23 (4 %)

**Fig. 1:**
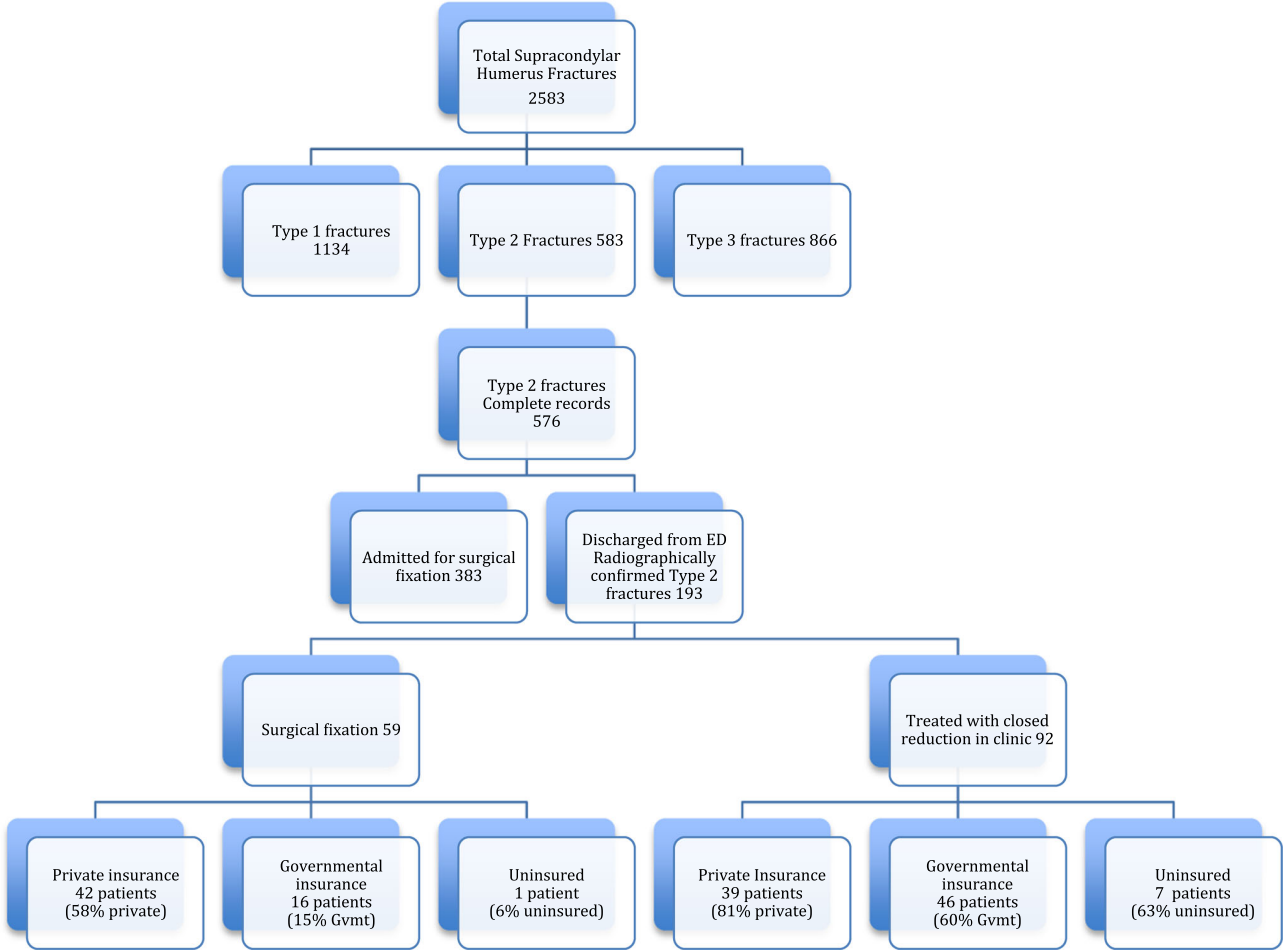
Flowsheet of care for 2583 supracondylar humerus fractures

Of the 2583 fractures, the 2000 patients with either type 1 fractures or type 3 fractures were excluded from the analysis as all type 3 fractures were admitted for surgery and all type 1 fractures were treated with closed management. Of the 583 type 2 fractures, 576 had complete records available for review (Fig. 1). 383 (66 %) patients were admitted at the initial encounter for surgery, and 193 (33 %) were discharged from the emergency department with plans for outpatient follow-up. All patients who were discharged were provided with phone numbers and addresses of all pediatric orthopedic providers in the city and told to follow up within the week. 88 (45 %) underwent a gentle closed reduction by a resident or nurse practitioner and long arm casting at 90° of flexion, as is the preferred initial treatment of certain surgeons in our center. There was no difference in the incidence of admission for immediate surgery between private and non-private insurance status amongst patients with type 2 fractures (OR 1.14 (95 % CI 0.81–1.63, *p* = 0.53)). Furthermore, neither patients with private insurance (OR 1.44 (95 % CI 0.66–3.12)) nor those with non-private insurance (OR 1.32 (95 % CI 0.60–2.89)) were more likely to have immediate surgery than those who were uninsured (*p* = 0.49).

Of the 193 patients who were discharged with documented type 2 fractures, 72 (37.3 %) had private insurance, 105 (54.4 %) had public insurance, and 16 (0.83 %) of these had no insurance. Of the 134 patients who did not eventually have surgery, 92 (68 %) were seen in one of our clinics. 61 % of those with non-private insurance returned to the clinic, compared to 81 % of the privately insured patients. The odds ratio of being seen if a patient had private vs non-private insurance was 2.39 (95 % CI 1.01–5.63, *p* = 0.04, Table 2). Forty-two patients did not follow up. Seven patients (5.2 %), all with non-private insurance, returned to the emergency department for cast removal due to an inability to secure a clinic visit. Four patients (3.0 %) were from out of state and were discharged with plans to follow up closer to their home. Thirty-one patients (16 %) were lost to follow-up. Ten patients (32.3 %) who were lost to follow-up had private insurance, while the remaining 21 patients (67.7 %) had non-private insurance. An attempt was made to contact the 31 patients who were lost to clinical follow-up by telephone, but only 10 (33 %) could be reached. Of these, 100 % stated that they had been unable to secure a clinic visit and therefore did not undergo surgical fixation of their fracture. Of the 31 who did not follow up in our system, 8 (25 %) were Spanish-speaking only, and only 8 (25 %) lived within the metropolitan area. The average distance from the hospital for the remaining 23 (75 %) was 36 miles. Patients who underwent a closed reduction in the emergency department were more likely to be seen for follow-up than those who were discharged without a closed reduction (OR 3.39 (95 % CI 1.43–7.98, *p* = 0.005)), although no formal bill for fracture care was generated at this time.

**Table 2:** Odds ratios of receiving any surgery, immediate surgery, postoperative follow-up, and delayed surgery for modified Gartland type II supracondylar humerus fractures

	Odds ratio (95 %CI) for patients with private insurance vs those with government insurance/uninsured	*p* value
Immediate surgery	1.14 (0.81–1.63)	0.45
Delayed surgery	2.46 (1.31–4.64)	0.01
Any surgery (immediate and delayed)	1.65 (1.12–2.45)	0.01
Seen for follow-up	2.39 (1.01–5.63)	0.04

Fifty-nine patients (30.6 %) ultimately underwent surgical fixation for either inadequate reduction or loss of reduction at follow-up. Of these, 42 patients were privately insured (58.3 % of the patients with private insurance), 16 had governmental insurance (15.1 %), and one was uninsured (6.3 %). There was no difference in time to delayed surgery between the groups (5.38 ± 0.64 days private vs 6.07 ± 1.07 non-private, *p* = 0.58). Patients with private insurance were 2.46 times more likely to have surgery than patients with public or no insurance (*p* = 0.005) (Table 2).

## Discussion and conclusion

Supracondylar humerus fractures are among the most common operative pediatric elbow injuries. Most authors agree that Gartland type III supracondylar humerus fractures should be addressed surgically within 24 h, and true Gartland type I fractures should be treated with casting alone [1, 12–16]. Controversy remains over a consensus approach to the treatment of Gartland type 2 supracondylar humerus fractures [4]. Many fractures of this type can be evaluated in the emergency department, splinted/casted, and discharged home to be followed in the clinic or to safely undergo operative treatment on an outpatient basis, avoiding hospitalization [8]. As the healthcare environment changes and outpatient procedures are further emphasized, there may be an impetus for treatment in this fashion, as it has been shown to be safe and reliable [4]. The aim of this study was to look at the subset of patients with a type II supracondylar humerus fracture who varied in their insurance coverage at the time of injury and to evaluate whether this played a part in the child receiving proper evaluation and treatment after discharge from the emergency department.

There is a large body of literature supporting the idea that children face limited access to care with Medicaid. Children insured under Medicaid who are in need of orthopedic care are no exception [17–19]. Sabharwal et al. reported on pediatric patients with fractures and noted that 52 % of patients with private insurance received orthopedic care, as compared to 22 % of the publicly insured patients [20]. Skaggs et al. surveyed 230 orthopedic practices and found that children with Medicaid insurance had limited access to orthopedic care, as 18 % (41/230) of offices would not see a child with Medicaid under any circumstances [21].

When considering the patients treated surgically at the time of initial presentation, our data suggest that payor status had no impact on the treatment decision. This was true for both type II and III fractures. Patients with public or no insurance who were discharged from the emergency department with plans to be seen in the clinic as an outpatient were 2.39 times less likely to be seen in the clinic and 2.46 times less likely to have resultant surgery than those with private insurance. Time to surgery for those patients who were discharged from the emergency department and were able to follow up was no different between privately insured patients and those with public insurance or those who were uninsured. This suggests that insurance status had no effect on the surgeon’s decision to pursue surgery once the patient was seen in the outpatient clinic. The issue appears to be the ability to secure an outpatient appointment.

The apparent impact of insurance status on patient follow-up may result in worse outcomes for patients, but the retrospective nature of this paper, which included a cohort of patients 3–7 years prior, prevented us from evaluating follow-up for 21 patients. It should be noted that only one patient without insurance who was discharged went on to have surgery. It is significant that most orthopedic practices in our city typically charge patients without insurance for initial consultation, and our hospital system often requires payment up front for surgical care if scheduled as an outpatient unless the patient qualifies for charity care. Immediate consultation and fracture care, both nonsurgical and surgical, can result in a bill for fracture care that allows subsequent follow-up to occur during the global billing period of fracture care. Patients may be seen regularly during this time without the need for additional surgeon’s fees, although additional radiographs may incur further charges. The upfront cost of surgery is not insignificant, and is typically too much of a financial burden for many families. Uninsured patients often obtain emergency Medicaid funding if they are admitted through the emergency department. Uninsured patients who are seen as an outpatient in the clinic often need to be admitted for surgery through the emergency department in order to bypass this barrier, adding further cost to the system.

The Emergency Medical Treatment and Active Labor Act (EMTALA) was enacted in 1986 to limit patient “dumping,” and requires that emergency departments provide care to patients within their capabilities, regardless of insurance status [22, 23]. The act does not address non-urgent medical conditions such as type II supracondylar humerus fractures, where outpatient care has been shown to be equivalent to immediate surgery as long as care is provided. It is generally frowned upon for surgeons to perform a “chart biopsy” and assess the patient’s insurance status before making a decision regarding care for a patient with a nonemergent condition, although it is certainly not illegal as long as care is not denied. The majority of the orthopedic literature regarding EMTALA centers around trauma and transfer to a level 1 center [24–26]. We are unaware of literature pertaining to EMTALA and pediatric orthopedics. While we are certainly not suggesting that all physicians screen patient charts to determine whether they need to be admitted or could be discharged with close follow-up, the current data do suggest that insurance status alone can impact the patient’s ability to achieve definitive care. As such, a treating physician should take into account the patient’s ability to follow up, and consideration should be given to providing surgical fixation at the initial point of care. This is especially relevant to most type II fractures at our institution, as they are treated with subsequent discharge being offered within a couple of hours of surgery. It is our feeling that a discussion of the patient’s insurance status should not be taboo if it is used to provide care for children who may not be able to follow up for outpatient care. Due to the rapid healing of supracondylar humerus fractures, delaying treatment for even a few weeks may result in a clinically significant malunion, as remodeling in these fractures is minimal [27–29].

As this was a retrospective analysis, our data do not account for surgeon preference regarding treatment of type II fractures. One of the four groups who treat children with supracondylar humerus fractures within our city prefers to treat the majority of extension type II fractures with closed reduction and cast immobilization in the emergency department and then close follow-up to determine the need for surgery, while the other practices typically favor planned delayed closed reduction and percutaneous pinning. Operative and nonoperative treatment are both supported in the literature [4, 5, 7–9, 13, 15, 30], although operative management is advocated by the AAOS [3]. While some may argue that surgical treatment is not required to manage these fractures due to satisfactory long-term results [31], the need for close follow-up is still important to avoid loss of reduction and malunion in either treatment method, as long-term angular deformity can result in symptomatic cubitus varus or loss of flexion [2, 27–29, 32–38]. As the focus of this study was on access to care, we did not review clinical outcomes, and the lack of a standardized clinical examination would make a retrospective review of patient charts incredibly challenging. Interestingly, patients who underwent a closed reduction in the ED were more likely to return for follow-up, perhaps suggesting that an interaction with an orthopedic surgeon helped direct the family to return for clinical evaluation. The retrospective nature of our study also limited our ablity to determine whether patients who did not receive follow-up actually contacted one of our offices, and what their clinical outcomes were. We contacted all four of the pediatric orthopedic offices in our city to evaluate the 31 patients who did not return for follow-up, and did manage to locate ten of these children. None of them ever obtained a surgical consultation. Our center is the only provider of pediatric surgical care in our region, but it is possible that some patients could have seen nonpediatric orthopedists for care. We suspect that it is improbable that patients without insurance would have been more likely to obtain surgical intervention at outside facilities, as our center provides the majority of indigent care in our region. Seven of the children who did not follow up, all with Medicaid insurance, returned to the emergency department for cast removal. While we cannot confirm that insurance status prevented these patients from following up, the data suggest that access to care is more difficult to obtain by patients with non-private insurance. It should also be noted that the decision not to carry medical insurance can be a personal one and not solely financially driven. Lack of insurance, while often related to financial means, is not exclusively driven by the ability to pay for an insurance plan.

In summary, insurance status was associated with access to outpatient surgical care, with privately insured patients being roughly 2.5 times more likely to have outpatient surgery than those with public or no insurance. Consideration should be given to evaluating the patient’s insurance status in order to provide the best and most appropriate care. If outpatient follow-up is unlikely given insurance limitations, then surgical management at the time of initial consult may be appropriate.
